# Ferns are less dependent on passive dilution by cell expansion to coordinate leaf vein and stomatal spacing than angiosperms

**DOI:** 10.1371/journal.pone.0185648

**Published:** 2017-09-27

**Authors:** Madeline R. Carins Murphy, Gregory J. Jordan, Timothy J. Brodribb

**Affiliations:** School of Biological Sciences, University of Tasmania, Hobart, Tasmania, Australia; University of Western Sydney, AUSTRALIA

## Abstract

Producing leaves with closely spaced veins is a key innovation linked to high rates of photosynthesis in angiosperms. A close geometric link between veins and stomata in angiosperms ensures that investment in enhanced venous water transport provides the strongest net carbon return to the plant. This link is underpinned by “passive dilution” via expansion of surrounding cells. However, it is not known whether this ‘passive dilution’ mechanism is present in plant lineages other than angiosperms and is another key feature of the angiosperms’ evolutionary success. Consequently, we sought to determine whether the ‘passive dilution’ mechanism is; (i) exclusive to the angiosperms, (ii) a conserved mechanism that evolved in the common ancestor of ferns and angiosperms, or (iii) has evolved continuously over time. To do this we first we assessed the plasticity of vein and stomatal density and epidermal cell size in ferns in response to light environment. We then compared the relationships between these traits found among ferns with modelled relationships that assume vein and stomatal density respond passively to epidermal cell expansion, and with those previously observed in angiosperms. Vein density, stomatal density and epidermal cell size were linked in ferns with remarkably similar relationships to those observed in angiosperms, except that fern leaves had fewer veins per stomata. However, plasticity was limited in ferns and stomatal spacing was dependent on active stomatal differentiation as well as passive cell expansion. Thus, ferns (like angiosperms) appear to coordinate vein and stomatal density with epidermal cell expansion to some extent to maintain a constant ratio between veins and stomata in the leaf. The different general relationships between vein density and stomatal density in ferns and angiosperms suggests the groups have different optimum balances between the production of vein tissue dedicated to water supply and stomatal tissue for gas exchange.

## Introduction

Efficient growth in vascular plants requires foliage that has stomatal valves in the epidermis to provide CO_2_ entry into the photosynthetic apparatus, and xylem veins to irrigate the desiccation-prone photosynthetic cells. These linked systems control water loss and water delivery to leaves, and are essential for the high rates of photosynthesis seen in vascular plants [1]. However, ferns typically pack substantially fewer veins into their leaves than their flowering plant relatives [2]. Along with other plant groups that evolved before the diversification of angiosperms, ferns have consistently produced leaves with low vein densities—around 2 mm mm^-2^—throughout 380 million years of evolution. By contrast, angiosperm leaves are endowed with vein densities > 5 mm mm^-2^, and up to 25 mm mm^-2^ [2]. This evolution of high vein densities in angiosperm leaves has been proposed to be a key innovation that enabled angiosperms to drastically increase their photosynthetic capacity and attain their current dominance—in terms of diversity and distribution—over other plant groups [3].

High vein density in leaves can permit high rates of photosynthesis by providing more water to irrigate the mesophyll. However, the association between water transport efficiency and CO_2_ assimilation [4, 5] is ultimately mediated by the relationship between veins and stomata [6]. This linkage has been recently demonstrated in angiosperms, in which the density of veins in leaf mesophyll tissue is tightly linked to the density at which stomata are present in leaf epidermis [7–9]. Furthermore, this link is mediated by the co-variance of both stomatal and vein density with epidermal cell size both among species, and within species in response to the light environment. According to the ‘passive dilution’ hypothesis, differential cell expansion dilutes veins and stomata in unison, maintaining a constant ratio between vein and stomatal densities and a balance between water supply (leaf hydraulic conductance [4, 5]) and transpirational demand (stomatal conductance) [7, 9]. This is thought to maximise the benefit-cost ratio of photosynthetic gain to plant investment in vein and stomatal infrastructure [6]. It has thus been suggested that the dramatic surge in vein density that characterised angiosperm diversification was accompanied by a proportional surge in stomatal density and decline in cell size [8]. Thus, a close geometric relationship between vein density, stomatal density and cell size, as well as plasticity in cell expansion, may be key features of angiosperm evolution. If so, this mechanism would be absent from earlier diverging plant groups, like the ferns. However, it has not yet been tested whether this ‘passive dilution’ mechanism acts as a coordinating mechanism across vascular plant lineages other than the angiosperms, with much lower vein densities.

Three scenarios are possible: either the ‘passive dilution’ mechanism is: (i) not present in the ferns because it is part of the unique suite of characteristics that facilitated angiosperm diversification; (ii) is found in both lineages because it is an ancient mechanism that evolved in a common ancestor and has been subsequently conserved; or (iii) is present in an intermediate form in the ferns because the developmental processes responsible for leaf functional architecture have continued to evolve over time. Evidence from the literature does not favour any one of these hypotheses. Some evidence suggests that ferns can adaptively co-regulate the ratio between vein and stomatal density [10, 11], but other research implies that water supply and demand may be mismatched in older plant lineages. For example, operational stomatal conductance tends to disagree with maximum theoretical stomatal conductance more in gymnosperms and ferns than in angiosperms [12]. This disparity may be the result of stomata not opening fully due to suboptimal water supply (i.e. vein densities may be too low to supply enough water to support the transpirational demand created by stomata). There are also reasons to suspect that different mechanisms may govern the coordination of stomata and veins in early and late-branching plant clades. The early evolution of ferns occurred under warm and relatively wet climates during the Palaeozoic and early Mesozoic [13, 14] at which time atmospheric CO_2_ concentrations are likely to have been considerably higher than those experienced during the diversification of the angiosperms [15, 16]. Thus, the diversification of these major plant groups occurred under vastly different climatic and atmospheric conditions which, in turn, may have led to different geometric relationships between veins, stomata and cell expansion. Furthermore, fern and angiosperm leaves develop in different ways. Many fern leaves undergo marginal development that typically results in a bifurcating vascular system lacking hierarchical vein orders [17–20], while angiosperms undergo diffuse leaf development and as a result can produce reticulate vascular systems with several vein orders [21–25]. Therefore, it is not known whether the current model of coordination between vein and stomatal density in angiosperms via their ‘passive dilution’ by cell expansion should apply to plant groups that diversified under different conditions in the deep past.

Thus, this study sought to determine whether the geometric vein, stomata and cell size relationships in early-branching vascular plants (ferns) are like the tightly linked relationships present in later-branching vascular plants (angiosperms). Therefore, testing whether the ‘passive dilution’ mechanism is exclusive to the angiosperms or has continued to evolve over time. To do this we first we assessed the plasticity of these traits in four ferns in response to light environment. We then compared relationships between vein density, stomatal density and epidermal cell size among a larger diversity of ferns (nine species) with modelled relationships that assume vein and stomatal density respond passively to epidermal cell expansion. These relationships among the ferns were next compared with previously identified relationships among a diverse range of angiosperm species.

## Materials and methods

### Plant material

Four fern species were selected initially based on the diversity of their preferred light habitats to test the plasticity of leaf traits to light environment [26–34] (Table 1). An additional five species were then selected to obtain a range in vein and stomatal densities that spanned the extremes in the fern clade. Veins and stomata are spaced in fern leaves at densities up to approximately 5 mm mm^-2^ [2] and 250 mm^-2^ [35], respectively. Hence, four species (*Astrolepis sinuata*, *Dryopteris cycadina*, *Pteridium esculentum* and *Todea barbara*) were grown in controlled conditions under contrasting light environments, with three plants per species grown under either full sun or shade. One individual of a further three species (*Cyrtomium macrophyllum*, *Drynaria quercifolia* and *Lygodium flexuosum*) was grown in full sun only. All plants in controlled conditions were grown in a mixture of 76% composted pine bark and 24% coarse potting sand and received weekly applications of liquid fertiliser (Aquasol, Hortico). They experienced day and night temperatures of 25 and 15°C, respectively, and ambient relative humidity. Natural light was supplemented by sodium vapour lamps in the morning and evening to maintain a 14-hour photoperiod. Plants grown in the full sun treatment received a maximum photosynthetic photon flux density (PPFD) of approximately 1800 μmol m^-2^ s^-1^ while plants in the shade treatment were grown under 90% shade cloth and received a maximum PPFD of approximately 200 μmol m^-2^ s^-1^. Two more species were sampled in the field. Leaf material was taken from an individual *Dicksonia antarctica* growing in full sun at the Sandy Bay campus of the University of Tasmania in Australia, and from an individual of *Dipteris conjugata* also growing in full sun on Mount Aoupinie in New Caledonia. These field species were added to extend the maximum end of the fern vein density spectrum. Species from the genus *Dipteris* exhibit the highest vein densities measured in ferns, but have never been successfully cultivated. Data from these species were compared with data from nine angiosperm species from a previous study grown under similar controlled sun and shade conditions [9].

**Table 1:** List of experimental species.

Species	Family	Native light habitat
*Astrolepis sinuata* (Lag. ex Sw.) D.M. Benham & Windham	Pteridaceae	Sun
*Cyrtomium macrophyllum* (Makino) Tagawa	Dryopteridaceae	Shade
*Dicksonia antarctica* Labill.	Dicksoniaceae	Shade
*Dipteris conjugata* Reinw.	Dipteridaceae	Sun
*Drynaria quercifolia* (L.) J. Sm.	Polypodiaceae	Sun
*Dryopteris cycadina* (Franch. & Sav.) C. Chr.	Dryopteridaceae	Shade
*Lygodium flexuosum* (L.) Sw.	Lygodiaceae	Both
*Pteridium esculentum* (G. Forst.) Cockayne	Dennstaedtiaceae	Both
*Todea barbara* T. Moore	Osmundaceae	Sun

### Stomatal conductance

Two healthy, mature leaves (fronds) from the most recently expanded cohort were selected per plant (*n* = 3) from the four ferns grown under both full sun and shade conditions to determine maximum stomatal conductance (mol m^-2^ s^-1^) using a Li-Cor Biosciences Li-6400 portable infrared gas analyser (Lincoln, NE, USA). Measurements were performed between 1000 and 1300 h when rates were expected to be maximal. Leaf chamber conditions were standardised during measurements. Leaf temperature was maintained at 25°C, CO_2_ concentration between 380 and 390 μmol mol^-1^, vapour pressure difference (VPD) at approximately 1.3 kPa and PPFD at 1000 μmol m^-2^ s^-1^.

### Pinna size and anatomical traits

The same leaves from the ferns grown under both full sun and shade conditions used to determine stomatal conductance were scanned at 300 pixels per inch (dpi) using a Canon CanoScan CS8800F flatbed scanner (Sydney, Australia) to measure pinna size (mm^2^). Three pinna were measured per leaf using the image analysis software ImageJ [36], and averaged to get the mean pinna size per leaf. Vein density (mm mm^-2^), stomatal density (mm^-2^) (all species were hypostomatic), stomatal size (mm^2^), stomatal index, epidermal cell density (total number of epidermal cells per unit area; mm^-2^) and epidermal cell size (mm^2^) were then quantified using paradermal sections taken from two locations on three pinna from each leaf (i.e. six sections per leaf). To prepare the paradermal sections the adaxial epidermis and palisade tissue were removed with a sharp razor and the remaining tissue placed in commercial household bleach (50 g L^-1^ sodium hypochlorite and 13 g L^-1^ sodium hydroxide) until all pigment was removed. Sections were then rinsed, stained with 1% toluidine blue and mounted on microscope slides in phenol glycerine jelly.

Entire sections were scanned at 1600 dpi to calculate vein density using ImageJ. This involved dividing the total length of all veins contained within a section by the section area. To capture the (sometimes) patchy distribution of stomata over the leaf surface stomatal density was sampled from all sections by photographing five fields of view along a transect at either 10 × objective magnification (field of view area 0.56 mm^2^), 20 × objective magnification (field of view area 0.14 mm^2^), or 20 × objective magnification through a 2.5 × tube (field of view area 0.025 mm^2^) using a Nikon Digital Sight DS-L1 camera (Melville, NY, USA) mounted on a Leica DM 1000 compound microscope (Nussloch, Germany). Different magnifications were used to maximise the precision of density counts and to ensure that approximately 20 stomata were counted per field of view. To quantify all other anatomical traits five fields of view were photographed from all sections at higher magnification. Photomicrographs were taken of sections from the sun and shade leaves of *D*. *cycadina* and *T*. *barbara*, the shade leaves of *P*. *esculentum* at 20 × objective magnification (field of view area 0.14 mm^2^) and from the sun and shade leaves of *A*. *sinuata* at 40 × objective magnification (field of view area 0.037 mm^2^) using the same microscope and camera. Additional photomicrographs were taken of sections from the sun leaves of *P*. *esculentum* at 40 × objective magnification through a 0.7 × tube (field of view area 0.073 mm^2^) using differential interference contrast with a Leica DFC450 digital microscope camera mounted on a Leica DM 2000 LED microscope to overcome the partial obscuration of epidermal cells by trichomes.

All leaf anatomical traits were quantified using ImageJ. Stomatal size was measured from five stomata (comprising a pair of guard cells) per field of view. Epidermal cell size (*S*_EC_) was calculated as:

$$S_{\text{EC}}=\frac{1-\left(D_{\text{S}}\times S_{\text{S}}\right)}{D_{\text{EC}}},$$ (1)

where *D*_S_ is stomatal density, *S*_S_ is stomatal size and *D*_EC_ is epidermal cell density. Stomatal index (SI) was calculated as:

$$\text{SI}=\left(\frac{D_{\text{S}}}{D_{\text{S}}+D_{\text{EC}}}\right)\times 100,$$ (2)

according to Salisbury [37]. Partial stomata and epidermal cells were included in density counts if visible along the top and right-hand border of photomicrographs and discarded if visible along the bottom and left-hand border.

Anatomical traits (vein density, stomatal density, stomatal size, stomatal index, epidermal cell density and epidermal cell size) were also measured for five additional fern species that were grown under full-sun conditions. One section was taken from an individual of each species to further examine vein-stomatal relationships in a larger sample of species. Vein density was quantified as described above, except in the case of *D*. *conjugata*, in which vein density was calculated from 10 fields of view photographed at 4 × objective magnification (field of view area 3.47 mm^2^). Stomatal density was quantified from 10 photomicrographs taken along a transect at either 10 or 20 × objective magnification, or at 40 × objective magnification through a 0.7 × tube (field of view area 0.073 mm^2^). All other anatomical traits were quantified from a further 10 photomicrographs taken at higher magnification: 20 × objective magnification in the case of *C*. *macrophyllum*, *D*. *conjugata*, *D*. *quercifolia* and *L*. *flexuosum* and 40 × for *D*. *antarctica*.

### Statistical analysis

The plastic response of vein density, stomatal density, stomatal index, epidermal cell size, pinna size and stomatal conductance to light intensity was assessed using two-way factorial analysis of variance (ANOVA) to examine the influence of light intensity and species on each of the traits listed above. Where interaction effects were significant, means for sun and shade plants were compared within species with unpaired t-tests corrected for multiple comparison using the Dunn–Sidak method [38]. Variation in leaf anatomy between ferns and angiosperms was also assessed using unpaired t-tests. Analysis of co-variance (ANCOVA) was used to test if a single linear regression could describe the relationship between vein density and √stomatal density among all ferns and a similar data set of nine angiosperms from a previous study. The same approach was used to compare the relationships between vein density and 1/√epidermal cell size and between stomatal density and 1/epidermal cell size among ferns and angiosperms. 1/√epidermal cell size, 1/epidermal cell size and √stomatal density was used according to the expectation that cell expansion dictates vein and stomatal density. The correlation coefficient (*r*^2^) and statistical significance (*P* < 0.05) of the co-variation between vein density and √stomatal density, vein density and 1/√epidermal cell size, stomatal density and 1/epidermal cell size, stomatal density and stomatal index, and stomatal conductance and vein density among ferns was then determined. For all analyses, log, square root, 1/(Y) or 1/√(Y) transformations were applied when needed to normalize the data. The relative contribution of 1/epidermal cell size and stomatal index to the *r*^2^ of the multiple regression in which they are predictors of stomatal density was quantified as a relative importance metric (lmg) using the ‘relimp’ package [39]. All analyses were performed in R [40].

### ‘Passive dilution’ models

**Fig 1:**
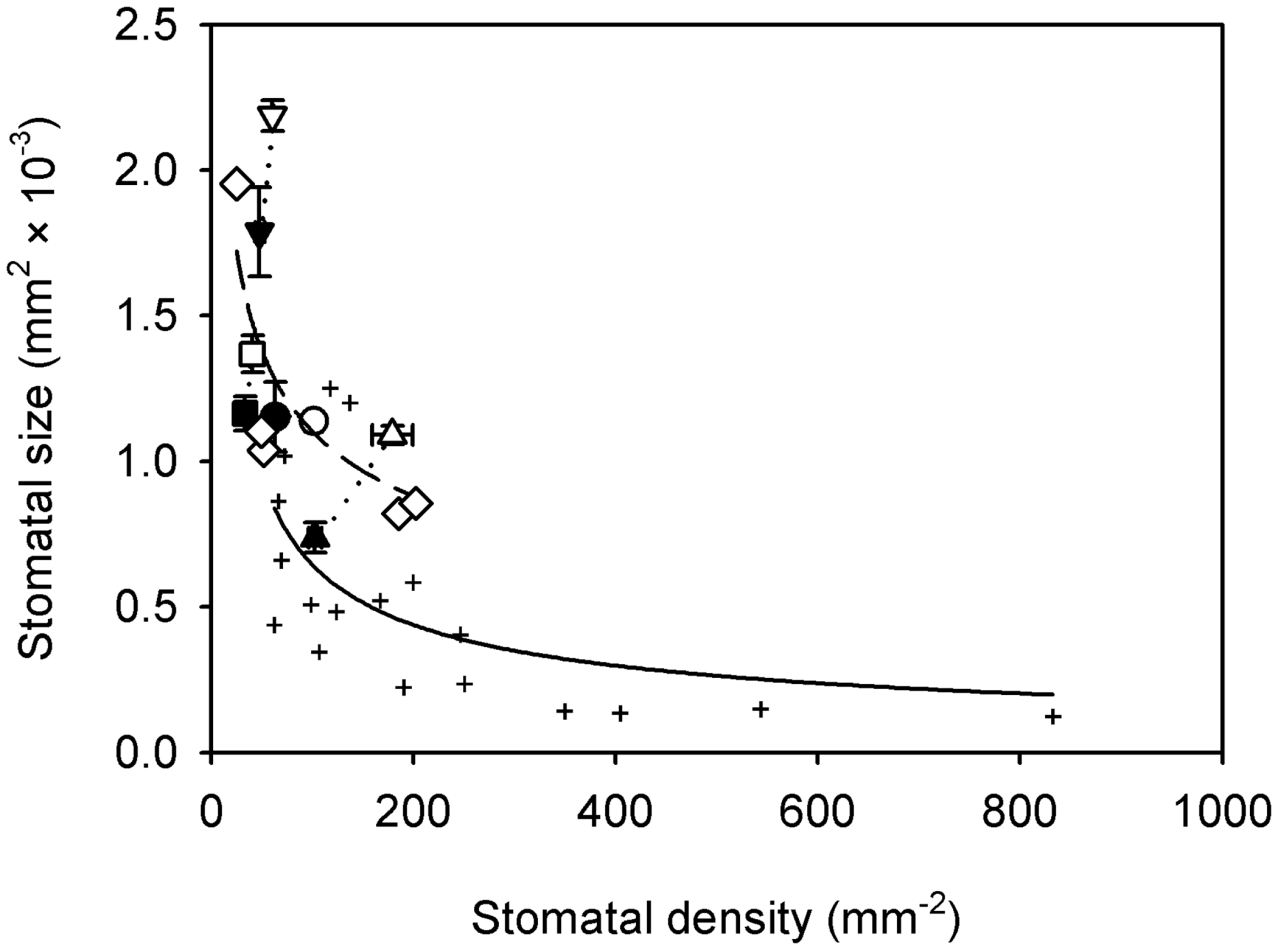
Relationship between stomatal size and density among ferns compared with angiosperms. Data points are means ± standard deviation of four fern species *Astrolepis sinuata* (circles), *Dryopteris cycadina* (squares), *Pteridium esculentum* (up-facing triangles) and *Todea barbara* (down-facing triangles) grown in sun (open symbols) and shade (closed symbols) (species pairs joined by a dotted black line), means of five additional ferns grown in sun only (diamonds) and means of nine angiosperm species (black crosses). Angiosperm data from Carins Murphy, Jordan [9]. A significant linear regression describes relationship among ferns when logarithms are taken of both variables (1/Y transformed data); log (*S*_S_) = log (-0.179) + 0.007 (log (*D*_S_) (*r*^2^ = 0.4, *F*_1,11_ = 7.31, *P* < 0.05) (dashed line). Untransformed data is shown. Solid black line is the best fit regression among the angiosperms (*r*^2^ = 0.37, *F*_1,16_ = 9.26, *P* < 0.01).

Relationships between vein density, stomatal density and epidermal cell size among ferns were compared with modelled relationships using ANCOVA performed in R. Modelled relationships were calculated according to the method outlined in Carins Murphy, Jordan [9]. Hence, they were based on the ‘passive dilution’ hypothesis under which vein and stomatal density are coordinated by the expansion of epidermal cells if vein and stomatal densities are uniquely related to epidermal cell size and the epidermis comprises only epidermal and stomatal cells with a constant ratio between them (stomatal index). Thus, epidermal cell size was calculated for a range of stomatal densities using a modified version of eq 1 substituting a × *D*s^b^ for *S*s, as follows:

$$S_{\text{EC}}=\frac{1-\left(D_{\text{S}}\times \left(a\times D_{\text{S}}{}^{b}\right)\right)}{\left(\frac{D_{\text{S}}}{\text{SI}}\right)-D_{\text{S}}},$$ (3)

using three different stomatal indices (the mean of all fern species and the mean ± 20%) and an empirical function based upon the pooled data from all ferns to account for the non-linear association between stomatal size and stomatal density (*a* = 0.0046 and *b* = -0.3159) (Fig 1). A negative correlation has been previously recognized between stomatal size and density [41].

The relationship between vein density and epidermal cell size was modelled using the simplifying assumption that vein length is associated with a fixed proportion of the perimeter of an epidermal cell. According to this assumption, one side of a theoretical square epidermal cell would always be in contact with vein tissue as it expanded. Thus, a geometric model of vein density as a function of epidermal cell size was determined for a fixed stomatal index (the mean of all fern species). Epidermal cell size was calculated as above. Assuming vein density (*D*_V_) was a function of epidermal cell size we fitted the function:

$$D_{\text{V}}=a\times S_{\text{EC}}^{-0.5},$$ (4)

where *a* is proportional to the epidermal cell perimeter associated with vein length. This returned a value of 0.1139 for *a*. This value was then used to predict the impact of epidermal cell size on vein density (using the equation: *D*_V_ = 0.1139 × *S*_EC_^-0.5^).

## Results

### Plasticity of fern leaf anatomy and morphology

The magnitude and direction that vein and stomatal density changed in response to sun and shade varied among the four ferns grown in contrasting light environments. Thus, the effect of light intensity on vein density varied substantially among species as indicated by a strong light intensity by species interaction effect (Tables 2 and 3). Shade induced a significant decrease in vein density in *P*. *esculentum*, a significant increase in *D*. *cycadina*, and had no significant effect on vein density in *A*. *sinuata* or *T*. *barbara* (Table 3). In contrast light intensity had a general effect on stomatal density and stomatal index, with the shade leaves of all species having lower stomatal densities and smaller stomatal indices than sun leaves. There was some variation in the size of the effects, as indicated by significant light intensity by species interaction effects that were much smaller than the main effects (Table 2). Pinna size, however, was not significantly affected by light intensity, and the effect of light intensity on epidermal cell size varied among species (Tables 2 and 3). Thus, the light intensity by species interaction effect on epidermal cell size was significant and like the main effect of light intensity (Table 2). Epidermal cells were significantly larger in the shade leaves of *A*. *sinuata* than in sun leaves but in all other species light-induced changes to epidermal cell size were non-significant (Table 3).

**Table 2:** Results of two-way ANOVA tests for the effect of light intensity on leaf traits in fern species.

	Source of variation:	Light intensity	Light intensity × Species
Trait:			
Epidermal cell size		***F***_**1,16**_ = **11.74, *P* < 0.01**	***F***_**3,16**_ = **3.99, *P* < 0.05**
Pinna size		*F*_1,16_ = 1.13, *P* > 0.05	*F*_3,16_ = 1.4, *P* > 0.05
Stomatal conductance		***F***_**1,16**_ = **178.74, *P* < 0.001**	***F***_**3,16**_ = **36.62, *P* < 0.001**
Stomatal density		***F***_**1,16**_ = **99.8, *P* < 0.001**	***F***_**3,16**_ = **5.76, *P* < 0.01**
Stomatal index		***F***_**1,16**_ = **85.7, *P* < 0.001**	***F***_**3,16**_ = **8.19, *P* < 0.01**
Vein density		***F***_**1,16**_ = **7.11, *P* < 0.05**	***F***_**3,16**_ = **13.47, *P* < 0.001**

*F*-values and *P*-values are given; bold text indicates *P* < 0.05.

**Table 3:** Epidermal cell size, pinna size, stomatal density, stomatal index, and vein density of leaves from sun and shade plants of four fern species (values are means ± standard error).

Species	Epidermal cell size (mm^2^ × 10^−3^)		*P*	Pinna size (cm^2^)		*P*	Stomatal density (mm^-2^)		*P*	Stomatal index (unitless)		*P*	Vein density (mm mm^-2^)		*P*
	Sun	Shade		Sun	Shade		Sun	Shade		Sun	Shade		Sun	Shade	
*Astrolepis sinuata*	1.8 ± 0.04	2.57 ± 0.17	**< 0.05**	1.56 ± 0.04	1.53 ± 0.17	1	101.53 ± 4.95	63.19 ± 1.91	**< 0.01**	19.52 ± 0.64	16.55 ± 1.49	0.45	3.63 ± 0.08	3.25 ± 0.14	0.29
*Dryopteris cycadina*	2.92 ± 0.04	3.21 ± 0.13	0.31	3.5 ± 0.23	2.65 ± 0.46	0.54	40.81 ± 2.63	33.60 ± 2.21	0.35	15.56 ± 0.77	13.05 ± 0.17	0.13	1.25 ± 0.04	1.52 ± 0.04	**< 0.05**
*Pteridium esculentum*	1.27 ± 0.06	1.7 ± 0.19	0.33	17.15 ± 5.2	22.04 ± 3.55	0.93	179.16 ± 11.64	102.79 ± 3.88	**< 0.05**	27.69 ± 1.11	17.98 ± 0.81	**< 0.01**	3 ± 0.07	2.36 ± 0.08	**< 0.05**
*Todea barbara*	3.55 ± 0.09	3.39 ± 0.23	0.95	12.53 ± 0.95	10.74 ± 1.41	0.82	60.51 ± 0.51	47.85 ± 2.67	**< 0.05**	20.77 ± 0.7	13.6 ± 0.46	**< 0.01**	1.15 ± 0.04	1.27 ± 0.10	0.81

Means of the two treatment groups were compared using unpaired *t*-tests. *P*-values < 0.05 are shown in bold.

### Relationships between veins, stomata, epidermal cells and stomatal index among ferns

**Fig 2:**
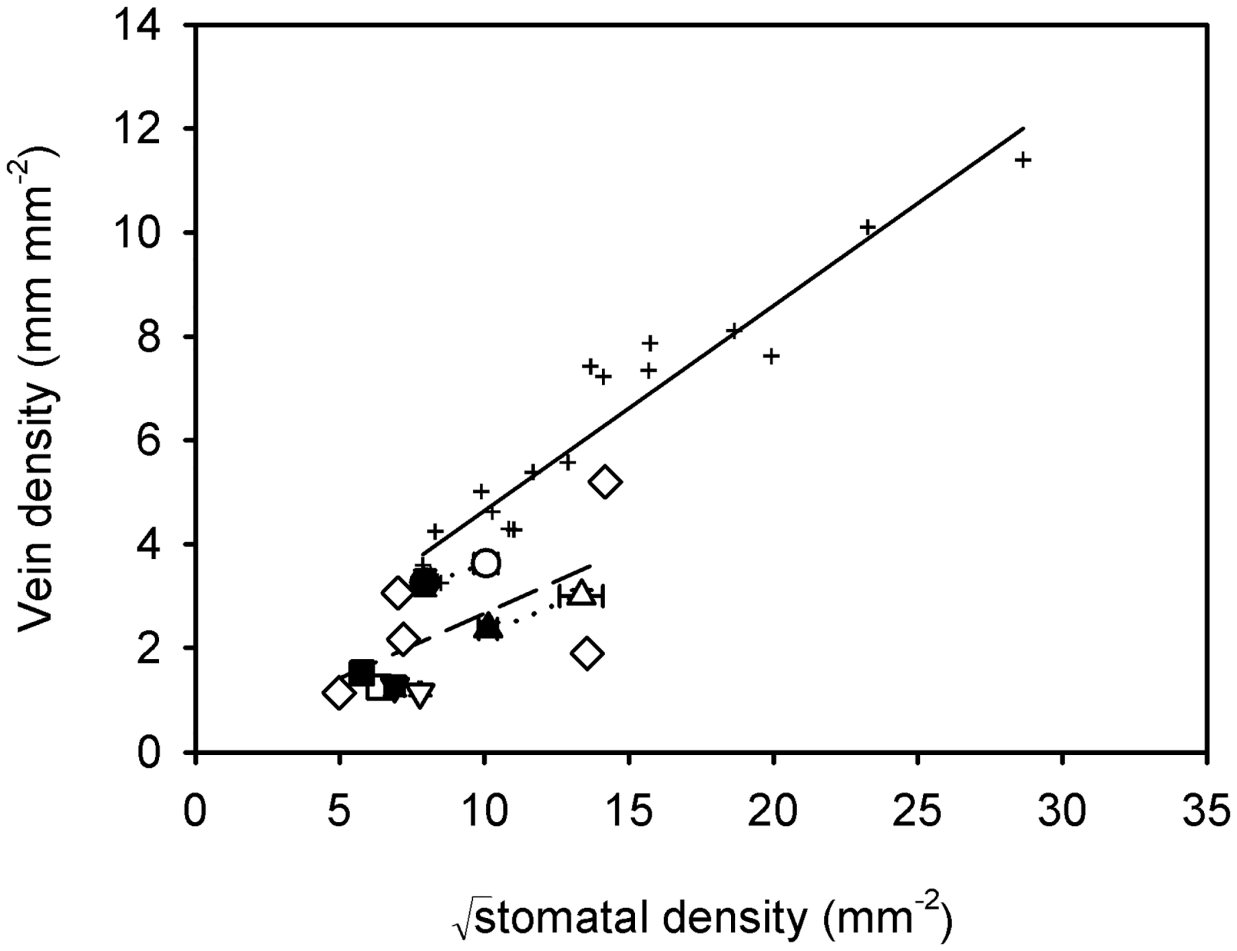
Relationship between vein and √stomatal density among ferns and among angiosperms. Data points are means ± standard deviation (symbols as in Fig 1). Angiosperm data from Carins Murphy, Jordan [9]. A significant linear regression describes the relationship between vein density and √stomatal density among ferns (log transformed data); *D*_V_ = 0.102 × √*D*_S_—0.156 (*r*^2^ = 0.41, *F*_1,11_ = 7.61, *P* < 0.05) (dashed line). Solid black line is the best fit regression among the angiosperms (*r*^2^ = 0.92, *F*_1,16_ = 186.3, *P* < 0.001).

**Fig 3:**
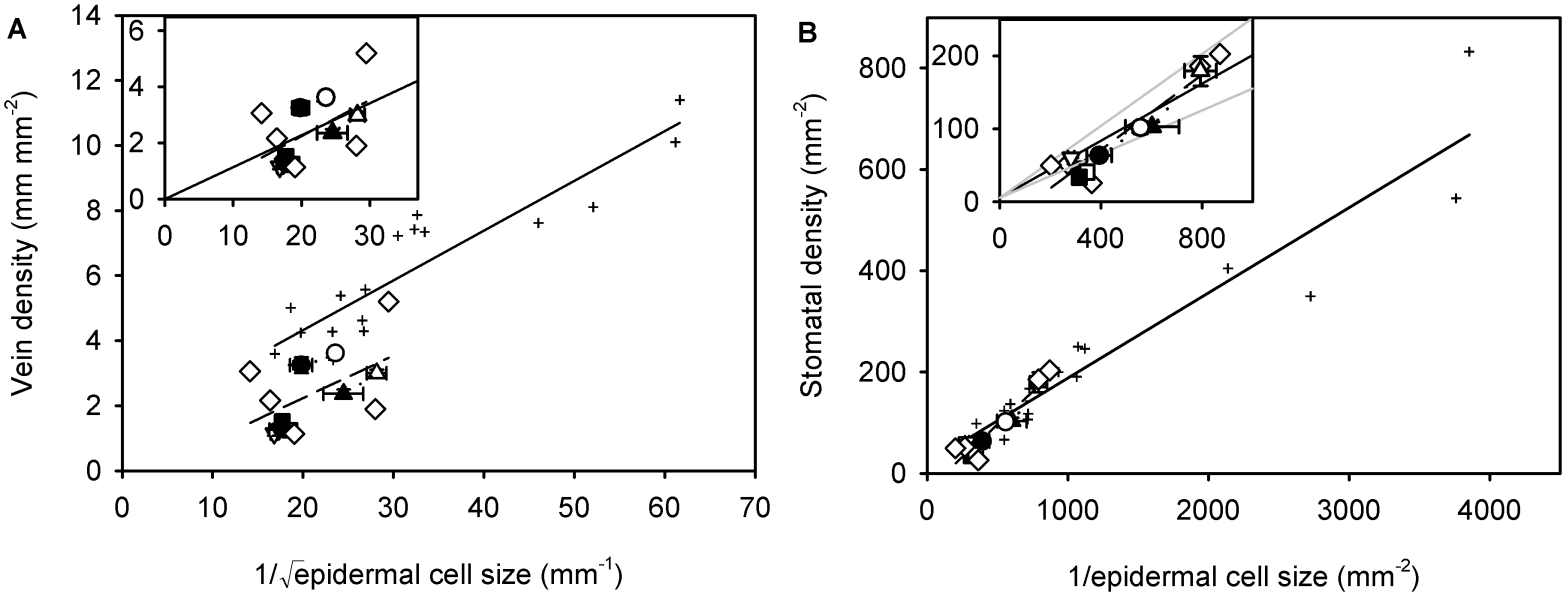
Relationships between vein density (*D*_V_) and 1/√epidermal cell size (*S*_EC_) [A] and stomatal density (*D*_S_) and 1/epidermal cell size [B] among ferns and among angiosperms. Data points are means ± standard deviation (symbols as in Fig 1). Angiosperm data from Carins Murphy, Jordan [9]. Inserts show relationships among ferns (dashed lines) compared with modelled relationships (solid lines). Modelled relationships assume that epidermal cell expansion drives vein and stomatal density and that stomatal index is constant. Black solid lines are models using the mean fern stomatal index and grey solid lines are models using the mean fern stomatal index ± 20% (see Materials and methods for details). The slope of the observed relationship between vein density and 1/√epidermal cell size was not significantly different from the slope of the modelled relationship while the slope of the observed relationship between stomatal density and 1/epidermal cell size was significantly different from the slope of the modelled relationship using the mean fern stomatal index. Significant linear regressions describe the relationships between vein density and 1/√epidermal cell size; *D*_V_ = 0.13 × 1/√*S*_EC_—0.359 (*r*^2^ = 0.3, *F*_1,11_ = 4.68, *P* = 0.05) and stomatal density and 1/epidermal cell size; *D*_S_ = 0.259 × 1/*S*_EC_—33.266 (*r*^2^ = 0.91, *F*_1,11_ = 110.4, *P* < 0.001) among the ferns.

**Fig 4:**
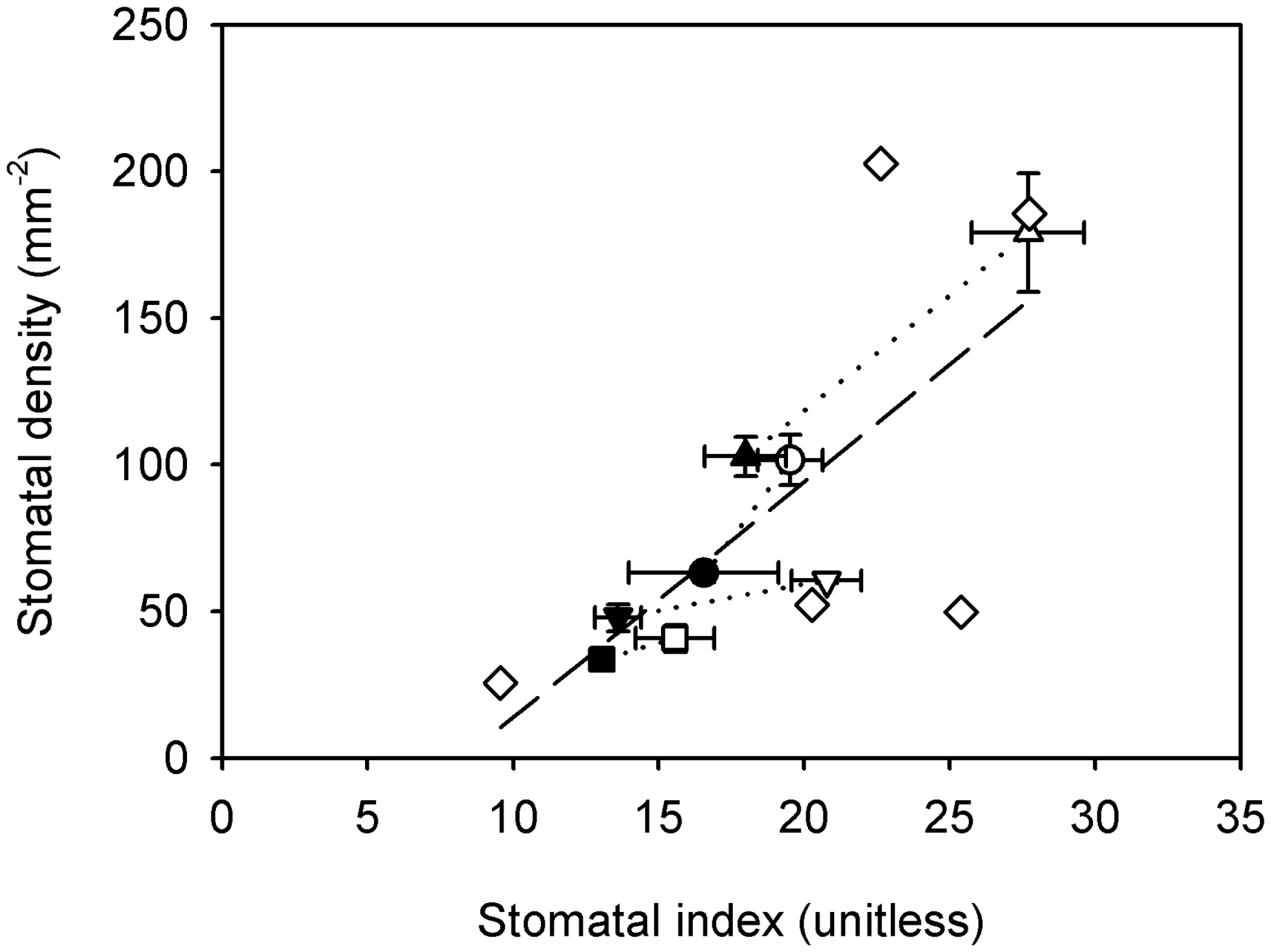
Relationship between stomatal density (*D*_S_) and stomatal index (SI) among ferns. Data points are means ± standard deviation (symbols as in Fig 1). The relationship is described by a significant linear regression (1/√(Y) transformed data); *D*_S_ = -0.006 × SI + 0.232; (*r*^2^ = 0.62, *F*_1,11_ = 18.25, *P* < 0.01).

There was a significant correlation between vein density and √stomatal density among ferns (*r*^2^ = 0.41, *F*_1,11_ = 7.61, *P* < 0.05) (Fig 2). Likewise, vein density was marginally significantly correlated with 1/√epidermal cell size among ferns (*r*^2^ = 0.3, *F*_1,11_ = 4.68, *P* = 0.05) (Fig 3A), and stomatal density was strongly correlated with 1/epidermal cell size (*r*^2^ = 0.91, *F*_1,11_ = 110.4, *P* < 0.001) (Fig 3B) and stomatal index (*r*^2^ = 0.62; *F*_1,11_ = 18.25, *P* < 0.01) (Fig 4).

Consistent with the ‘passive dilution’ hypothesis, modelling accurately described the observed relationship between vein density and 1/√epidermal cell size (insert in Fig 3A and Table 4). However, as would be predicted from the strong relationship between stomatal density and stomata index among ferns, the modelling did not fully describe the relationship between stomatal density and 1/epidermal cell size (insert in Fig 3B and Table 4). Specifically, the slope and intercept of the observed relationship between vein density and epidermal cell size was not significantly different from the modelled relationship; while in contrast, the slope of the observed relationship between stomatal density and epidermal cell size was significantly different from the modelled relationship using the mean stomatal index of all ferns. The slope of the observed relationship between stomatal density and 1/epidermal cell size was 32.2% greater than the slope of the modelled relationship. Despite this, 1/epidermal cell size was a more important determinant of stomatal density than stomatal index as it explained 66% of the variation in stomatal density compared with the 34% explained by stomatal index.

**Table 4:** Results of ANCOVA tests to determine whether observed relationships are explained by modelled relationships.

	Source of variation:	Covariate	Relationship type	Covariate × relationship type
Trait pair:				
Stomatal density and 1/epidermal cell size		***F***_**1,20**_ = **390.86, *P* < 0.001**	*F*_1,20_ = 2, *P* > 0.05	***F***_**1,20**_ = **7.48, *P* < 0.05**
Vein density and 1/√epidermal cell size		***F***_**1,21**_ = **30.02, *P* < 0.001**	*F*_1,21_ = 0.04, *P* > 0.05	*F*_1,20_ = 0.11, *P* > 0.05

*F*-values and *P*-values are given; bold text indicates *P* < 0.05.

### Relationship between stomatal conductance and vein density among ferns

**Fig 5:**
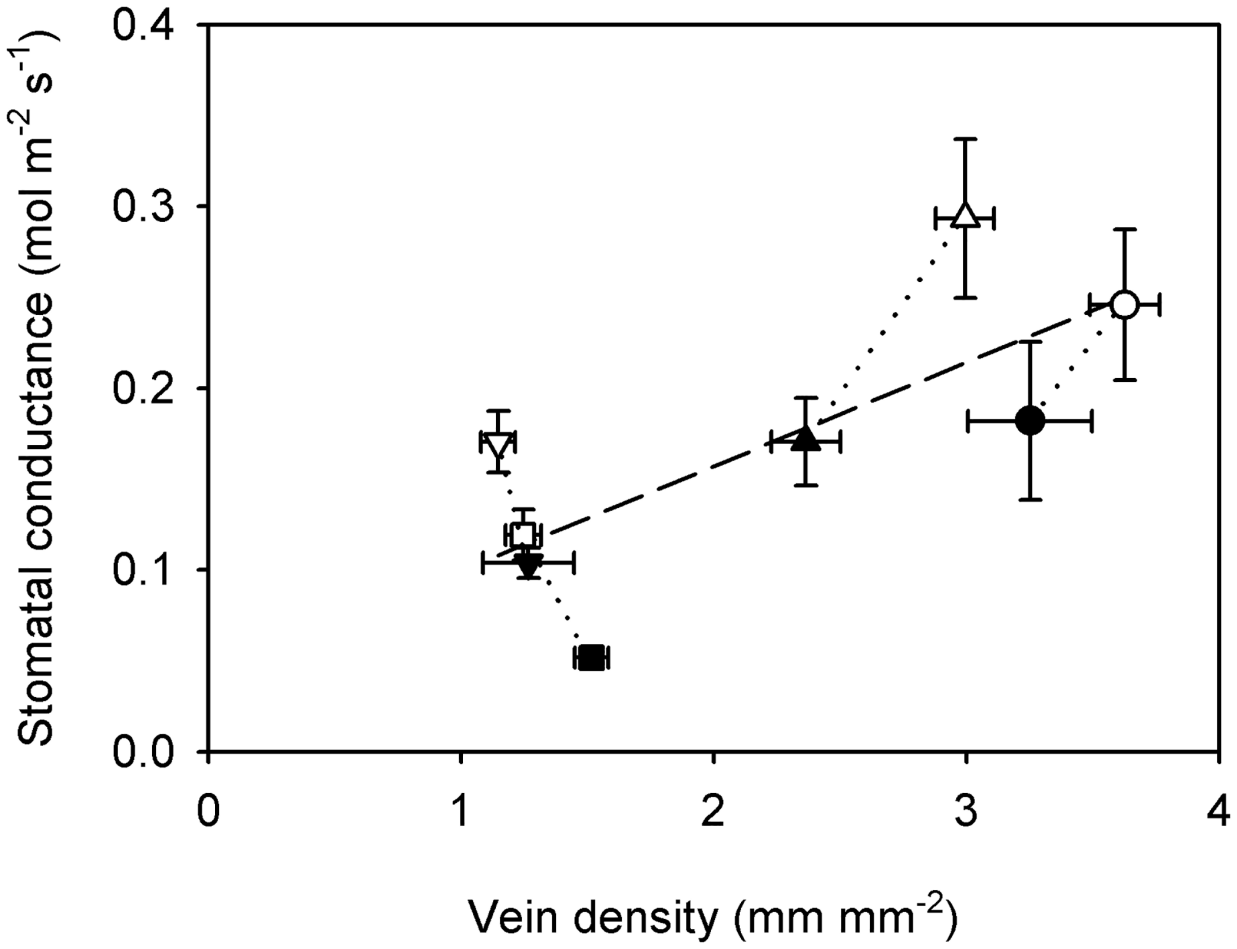
Relationship between stomatal conductance (*g*_S_) and vein density (*D*_V_) among ferns. Data points are means ± standard deviation (symbols as in Fig 1). A significant regression describes the relationship between stomatal conductance and vein density among ferns; *g*_S_ = 0.0572 × *D*_V_ + 0.0426 (*r*^2^ = 0.56, *F*_1,6_ = 7.52, *P* < 0.05) (dashed line).

Light intensity had a general effect on vapour-phase water conductance in ferns, with shade leaves having lower stomatal conductance than sun leaves. Thus, the main effect of light intensity on stomatal conductance was very large and significant (Table 2). There was some variation in the size of the effect, as indicated by a significant light intensity by species interaction effect that was much smaller than the main effects (Table 2). Furthermore, there was a significant positive relationship between stomatal conductance and vein density among ferns (*r*^2^ = 0.56, *F*_1,6_ = 7.52, *P* < 0.05; Fig 5).

### Relationships between veins, stomata and epidermal cells in ferns compared with angiosperms

**Fig 6:**
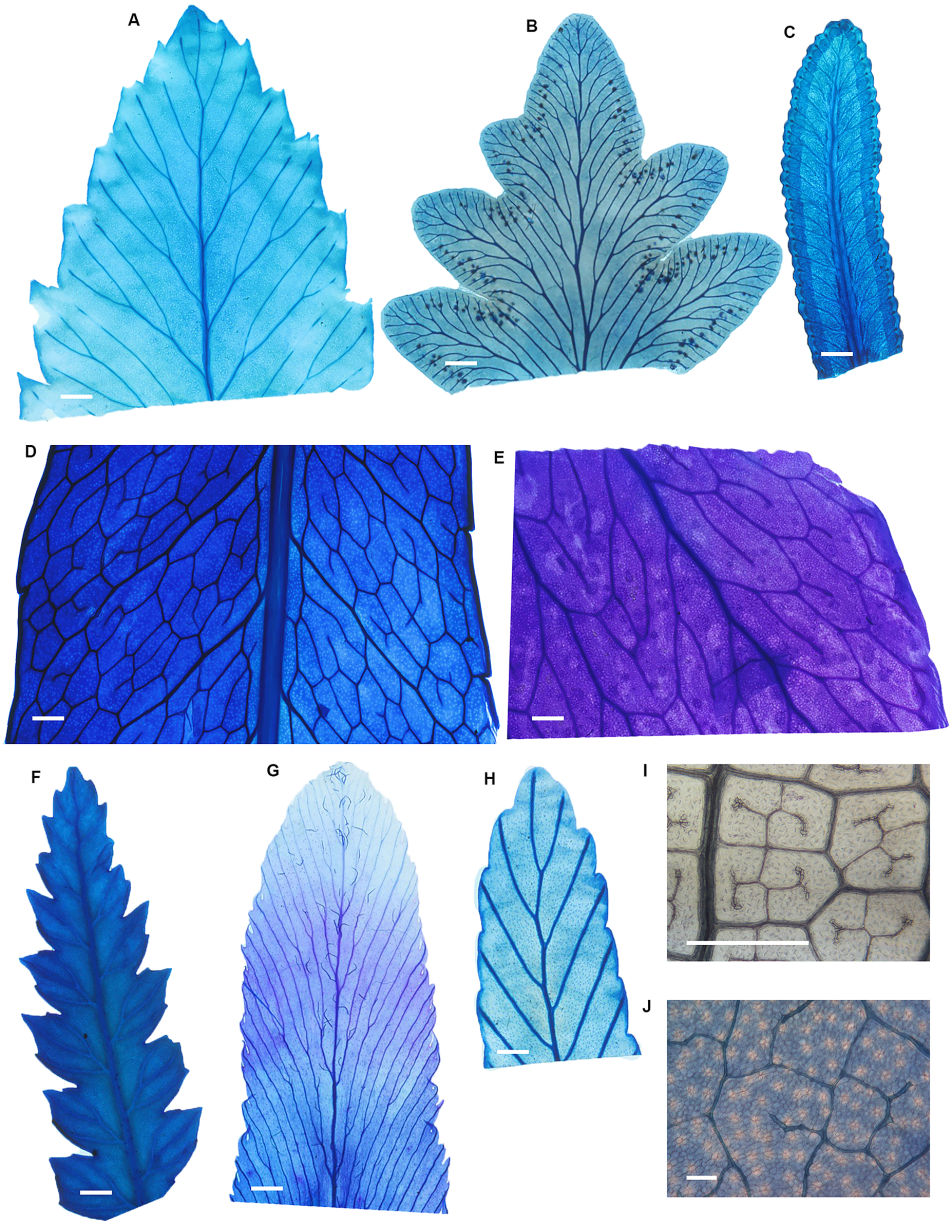
The vascular architecture in the leaves of experimental ferns and a representative angiosperm. *Dryopteris cycadina* [A], *Astrolepis sinuata* [B], *Pteridium esculentum* [C], *Drynaria quercifolia* [D], *Cyrtomium macrophyllum* [E], *Dicksonia antarctica* [F], *Lygodium flexuosum* [G], *Todea barbara* [H], *Dipteris conjugata* [I] and *Solanum laciniatum* (angiosperm) [J]. Scale bars are 1 mm for all ferns and 0.1 mm for the angiosperm.

**Fig 7:**
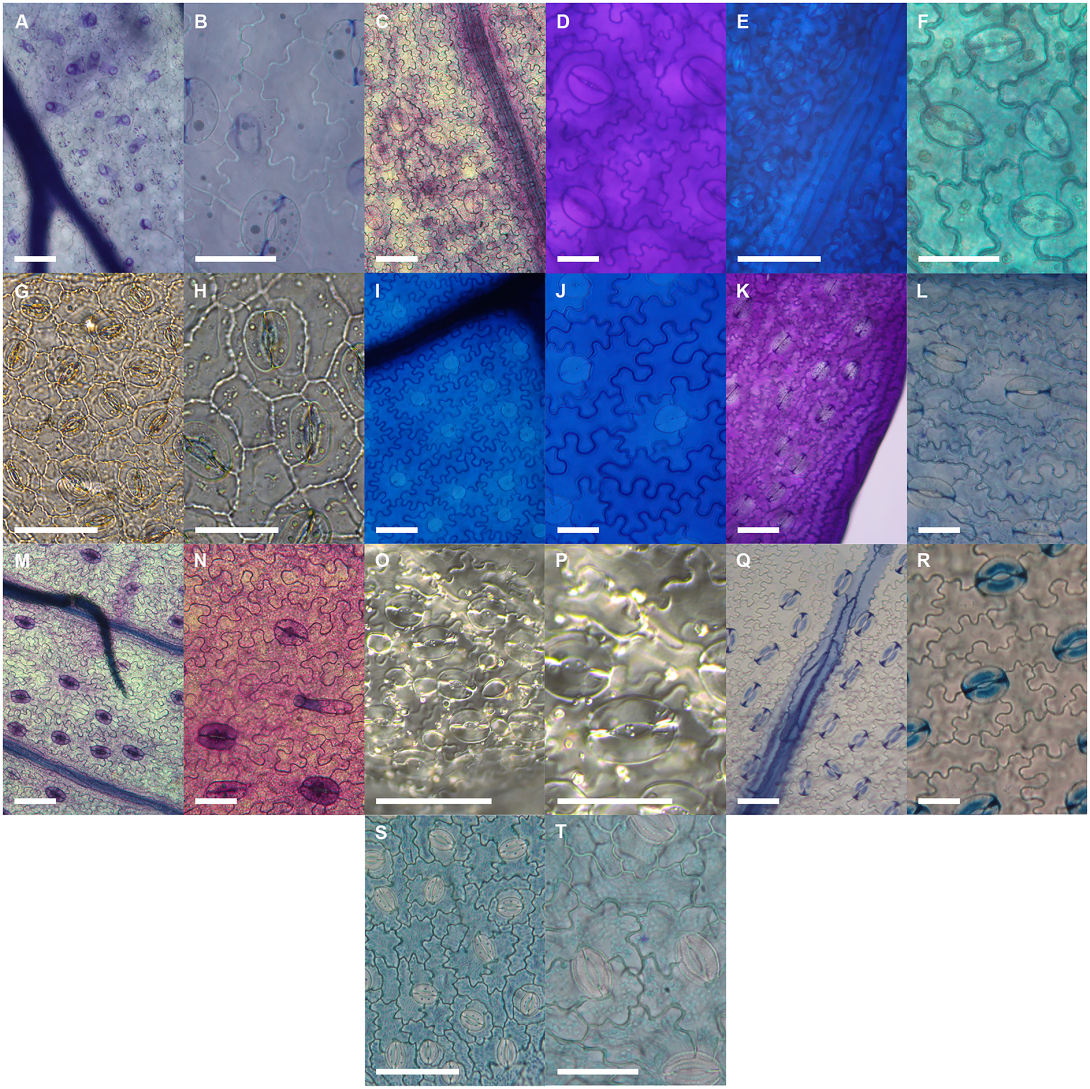
The size and arrangement of stomata and epidermal cells in the leaves of experimental ferns and a representative angiosperm. *Astrolepis sinuata* [A and B], *Cyrtomium macrophyllum* [C and D], *Dicksonia antarctica* [E and F], *Dipteris conjugata* [G and H], *Drynaria quercifolia* [I and J], *Dryopteris cycadina* [K and L], *Lygodium flexuosum* [M and N], *Pteridium esculentum* [O and P], *Todea barbara* [Q and R] and *Solanum laciniatum* (angiosperm) [S and T]. Scale bars are 0.1 mm for low magnification images and 0.05 mm for high magnification images.

Compared with angiosperms, ferns had leaves with larger epidermal cells (*P* < 0.01) and lower densities of veins (*P* < 0.001) and stomata (*P* < 0.05) (Figs 6 and 7). Slopes that describe the relationships between vein density and √stomatal density and between vein density and 1/√epidermal cell size among ferns were not significantly different from slopes describing the same relationships among nine angiosperms (Figs 2 and 3A and Table 5). The intercepts for both relationships among the ferns, however, were significantly different from the intercepts for the same relationships among angiosperms. Thus, fern leaves had less vein length for a given density of stomata or epidermal cells than angiosperm leaves. In comparison, the slope describing the relationship between stomatal density and 1/epidermal cell size among the fern species was slightly steeper (53.9%) than the slope of the same relationship among the angiosperms (Fig 3B and Table 5).

**Table 5:** Results of ANCOVA tests to determine whether observed relationships among ferns are like those among angiosperms.

	Source of variation:	Covariate	Plant group	Covariate × plant group
Trait pair:				
Stomatal density and 1/epidermal cell size		***F***_**1,27**_ = **295.21, *P* < 0.001**	*F*_1,27_ = 3.76, *P* > 0.05	***F***_**1,27**_ = **15.32, *P* < 0.001**
Vein density and 1/√epidermal cell size		***F***_**1,28**_ = **166.68, *P* < 0.001**	***F***_**1,28**_ = **27.89, *P* < 0.001**	*F*_1,27_ = 0.15, *P* > 0.05
Vein density and √stomatal density		***F***_**1,28**_ = **249.23, *P* < 0.001**	***F***_**1,28**_ = **31.17, *P* < 0.001**	*F*_1,27_ = 3.08, *P* > 0.05

*F*-values and *P*-values are given; bold text indicates *P* < 0.05.

## Discussion

Our results show that although anatomical responses to light in our fern sample were less predictable than in woody angiosperms, the geometric relationships between veins, stomata and epidermal cells among the ferns included in this study were remarkably similar to those previously observed among a diverse range of angiosperms [9]. This means that the low densities of stomata and veins in ferns are associated with larger epidermal cells in both ferns and angiosperms. However, a significant difference in the ratio of veins to stomata, and more active control of stomatal differentiation in the ferns compared with angiosperms suggests that the developmental processes responsible for leaf functional architecture have continued to evolve during fern and angiosperm diversification. Despite this difference, a fairly constant ratio between vein and stomatal density still appears to be maintained in both fern and angiosperm leaves.

### Active as well as passive processes contribute to vein and stomatal frequency in ferns

Geometric relationships between veins, stomata and epidermal cells observed in the ferns from this study are similar to, but not the same as, those previously observed in angiosperms [9]. The slope of the relationship between stomatal density and epidermal cell size among ferns is steeper than the slope of the same relationship among angiosperms. Interestingly, although modelled and observed relationships between vein density and both stomatal density and epidermal cell size across ferns and angiosperms are described by the same slopes, the intercepts of both relationships are lower in ferns than in angiosperms. A similar off-set was reported by authors who examined the relationship between vein and stomatal density in one fern species (*Cyathea australis*) and four angiosperms [11]. This indicates that ferns produce less vein length per stomata or epidermal cell than angiosperms. A smaller investment in veins may be linked to the lower maximum stomatal porosity of ferns compared with angiosperms [42]. Despite the generally large size of fern stomata, small maximum apertures mean that ferns may have intrinsically lower stomatal conductances than angiosperms for the same stomatal density [12].

Thus, ferns appear to maintain a fairly constant ratio between vein and stomatal density in part by the same ‘passive dilution’ mechanism as angiosperms, but with a different ratio between veins and stomata. However, the significant correlation between stomatal density and index among ferns suggests that stomatal density is actively controlled by changes to the rate of stomatal differentiation as well as passively by epidermal cell expansion. Thus, the frequency at which stomata differentiate (reflected in the stomatal index) is likely modified independently of changes to epidermal cell size and vein density.

### Limited cell size plasticity in ferns may restrict passive modification of vein and stomatal density

The fern species studied here had a fairly limited capacity to make plastic changes to vein and stomatal frequency and epidermal cell size in response to light environment. In angiosperms, large changes to cell size are associated with significant changes in vein and stomatal density [9, 43]. Hence, ferns may rely more heavily on active adjustment of stomatal density (stomatal differentiation) because they have a limited capacity to manipulate cell size. Not only would this potentially reduce the coordination between vein and stomatal density within species it may also place an upper limit on the rates of gas exchange achievable by ferns because they may be unable to reduce cell sizes enough to produce high densities of veins and stomata. This premise is supported by the generally low vein densities observed in fern leaves compared with angiosperm leaves [2]. The typically larger genomes of ferns compared with angiosperms [44, 45] may limit their cell size plasticity. Given the fundamental correlation between genome size and cell size [46], large genomes may limit the minimum sizes of fern cells. In this study, the epidermal cells of ferns tended to be larger, and veins and stomata more widely spaced than in angiosperms. Although genome size was not measured, *T*. *barbara* is reported to have a genome size (1C) of 21.01 pg and *Pteridium* and *Dryopteris* species have genomes that range in size from approximately 8 to 16 pg and 6 to 24 pg, respectively [47]. Whereas the genomes of angiosperms are often smaller than 3.5 pg [44]. For example, four of the angiosperms used in this study had genomes that ranged in size from 0.6 to 4.93 pg [48].

### Differences in leaf development may contribute to the weaker link between stomatal density and epidermal cell expansion in ferns compared with angiosperms

Stomata and epidermal cells may be more loosely linked geometrically in ferns compared with angiosperms because of general differences in leaf development between clades. Most fern leaves undergo marginal development which generally results in bifurcating vascular systems that lack hierarchical vein orders [17–20], while angiosperms undergo diffuse leaf development and can produce reticulate vascular systems with many vein orders [21–25]. The strong link between vein density, stomatal density and epidermal cell size seen in angiosperms suggests that most veins and stomata are differentiated before the completion of leaf expansion [49]. In contrast, the partial independence of stomatal density from epidermal cell size observed in ferns suggests that stomatal differentiation may continue for longer during leaf expansion. Therefore, marginal leaf development in ferns may reduce the degree of coordination between vein and stomatal density in ferns, and also potentially result in a less homogenous distribution of stomata throughout the leaf epidermis and a subsequent decrease in overall rates of CO_2_ uptake.

## Conclusions

Relationships between vein density, stomatal density and epidermal cell size among ferns are very similar to those observed among angiosperms. However, there is little plasticity in these traits within ferns species, fern leaves have less vein length per stomata than angiosperms and changes to stomatal density are actively regulated by stomatal differentiation, as well as epidermal cell expansion. Despite this, epidermal cell size is a strong determinant of vein and stomatal density in ferns (explaining 66% of the variation in stomatal density versus 34% explained by stomatal index). Thus, ferns (like angiosperms) appear to use the co-variance of vein and stomatal density with epidermal cell expansion to some extent to maintain a constant ratio between the abundance of veins and stomata in the leaf. The different general relationships between vein density and stomatal density in ferns and angiosperms suggests that these groups have different conservative optimum balances between the production of vein tissue dedicated to water supply and stomatal tissue for gas exchange.

## Supporting information

S1 Appendix Table of data used for analyses. (XLSX)
